# IgA: Structure, Function, and Developability

**DOI:** 10.3390/antib8040057

**Published:** 2019-12-05

**Authors:** Patrícia de Sousa-Pereira, Jenny M. Woof

**Affiliations:** 1 School of Life Sciences, University of Dundee, Dundee DD1 5EH, UK; 2 CIBIO-InBIO, Campus Agrário de Vairão, University of Porto, 4485-661 Vairão, Portugal

**Keywords:** immunoglobulin A, IgA, structure, FcαRI, CD89, immune evasion, therapeutic antibodies

## Abstract

Immunoglobulin A (IgA) plays a key role in defending mucosal surfaces against attack by infectious microorganisms. Such sites present a major site of susceptibility due to their vast surface area and their constant exposure to ingested and inhaled material. The importance of IgA to effective immune defence is signalled by the fact that more IgA is produced than all the other immunoglobulin classes combined. Indeed, IgA is not just the most prevalent antibody class at mucosal sites, but is also present at significant concentrations in serum. The unique structural features of the IgA heavy chain allow IgA to polymerise, resulting in mainly dimeric forms, along with some higher polymers, in secretions. Both serum IgA, which is principally monomeric, and secretory forms of IgA are capable of neutralising and removing pathogens through a range of mechanisms, including triggering the IgA Fc receptor known as FcαRI or CD89 on phagocytes. The effectiveness of these elimination processes is highlighted by the fact that various pathogens have evolved mechanisms to thwart such IgA-mediated clearance. As the structure–function relationships governing the varied capabilities of this immunoglobulin class come into increasingly clear focus, and means to circumvent any inherent limitations are developed, IgA-based monoclonal antibodies are set to emerge as new and potent options in the therapeutic arena.

## 1. Introduction

The human immune system expends a considerable amount of energy in production of immunoglobulin A (IgA), since more IgA is made than all the other classes of immunoglobulin (Ig) combined. IgA is present in both serum, where at 2–3 mg/mL it is the second most prevalent circulating Ig after IgG, and in external secretions such as those that bathe mucosal surfaces, where it is the predominant Ig. It has been calculated that around 60 mg of IgA is produced per kilogram of body weight per day in the average human [1,2], much of it being localised at mucosal surfaces. Such surfaces, which collectively have a surface area in adult humans of around 400 m^2^ [3], are major sites of vulnerability, given their exposure to the environment, and IgA clearly plays a critical role in their protection against attack by invading pathogens.

In humans, there are two subclasses of IgA, named IgA1 and IgA2. Like all Ig, each subclass comprises a basic molecular unit of two identical heavy chains (HCs) and two identical light chains (LCs). Each chain begins at its N-terminus with a variable region, which is followed by a constant region. The LCs are the same in each subclass, but the HCs differ within their constant regions, which are encoded by distinct Cα genes. Two allotypic variants of human IgA2, known as IgA2m(1) and IgA2m(2), have been characterised. A third IgA2 variant, termed IgA2(n), has been described [4], but while presumed to be an allelic form, its penetrance in the population remains to be investigated.

Unlike other Ig classes, IgA exists in multiple molecular forms. In human serum, the predominant IgA form is monomeric, i.e., comprises 2HC and 2LC, with a subclass distribution of about 90% IgA1 and 10% IgA2. In contrast, the main molecular form found at mucosal surfaces, known as secretory IgA (SIgA), is dimeric, although some higher molecular weight species, including trimers and tetramers, are also present. Here the relative proportion of the two subclasses is more closely matched; an average distribution being about 40% IgA1 and 60% IgA2, though this varies depending on the particular mucosal site sampled.

Genetic sequence analysis has confirmed the presence of IgA in all categories of mammals (placental, marsupials, and monotremes) and in birds. However, there are notable species differences. Most mammals have a single IgA isotype. IgA1 and IgA2 subclasses akin to those in humans are only present in related primates, including chimpanzees, gorillas, and gibbons [5], consistent with IgA1 arising relatively recently in evolutionary terms. Orangutans have an equivalent of IgA1, but appear to have lost their form of IgA2. The other group of mammals to have more than one IgA are rabbits and other lagomorphs, which have a massively expanded number of IgA genes, resulting in 14 known subclasses, 11 of which are expressed. A 15th IgA was recently described in domestic European rabbits [6]. While IgA is known to play a common role in protection at mucosal surfaces [7], the levels, forms, and distribution of IgA vary. For example, in species commonly used in experimental research, including mice, rats, and rabbits, the main form of IgA in serum is dimeric rather than the monomeric form seen in humans. In these same species, unlike humans, the main source of IgA in the gut lumen is from bile. Another species difference relates to the prevalent Ig found in colostrum and milk. While in humans this is IgA, in cows, sheep, goats, and horses, the main immunoglobulin isotype present is IgG. 

Such species differences have tended to constrain research on the general features of IgA, and mean that there are inherent problems with extrapolation of results on IgA from animal models to humans. This review will focus primarily on human IgA, and will explore structure and function relationships and the prospect for developing IgA-based therapeutic monoclonal antibodies (mAb). The issue of species differences within the IgA system remains of relevance, given the growing interest in IgA as a potential therapeutic option and the requirement for meaningful models to robustly assess capabilities in this context.

## 2. IgA structure

### 2.1. General Features

In common with other Igs, both the HCs and LCs of IgA are folded into a number of variable (V) and constant (C) domains, each encoded by a separate exon. These number four in the HC (namely VH, Cα1, Cα2, and Cα3, starting from the N-terminus) and two in the LC (namely VL and CL, from the N-terminus). Each domain folds into a similar globular secondary structure, known as the immunoglobulin fold, a feature of all Igs. Typically stretching some 110 amino acids, each domain comprises two β-sheets made up of anti-parallel β-strands, which sandwich together around a stabilising disulphide bond.

Interposed between the Cα1 and Cα2 domains of each HC lies a flexible hinge region, which is particularly extensive in human IgA1 but shorter in human IgA2. Indeed, the hinge is the region of greatest difference between the two subclasses. Unlike IgG, there are no interchain disulphide bridges within the hinge region, which presumably affords the IgA hinge sequences, particularly the longer ones of IgA1, the ability to flex independently of each other, but may also increase the susceptibility to proteolysis.

**Figure 1:**
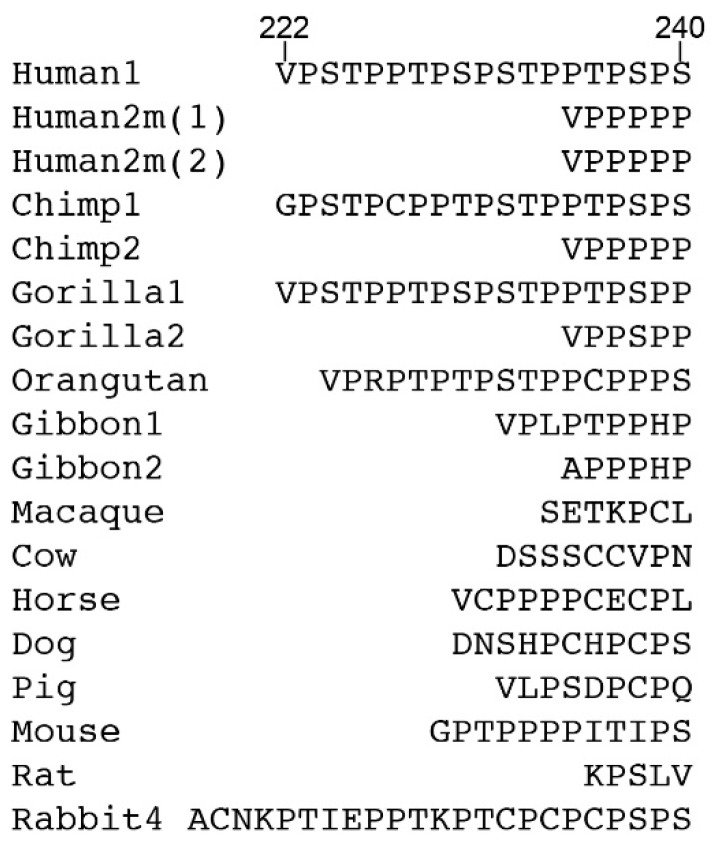
Hinge sequences of IgAs from different species. Numbers following the species name indicate the IgA subclass, and allotype where appropriate. Amino acid numbering above human IgA1 is according to the commonly adopted scheme used for IgA1 Bur [8].

The hinge of IgA1, rich in proline, serine, and threonine, contains a sequence missing in IgA2 that comprises two eight amino acid repeats (Figure 1). The hinge in human IgA is encoded in a sequence present at the 5′ end of the exon encoding the Cα2 domain, rather than by a separate exon or exons as seen for IgG. As in other Igs, the hinge affords flexibility to the whole IgA molecule that is critical for activity. It varies considerably in length and sequence between IgAs from different species (Figure 1).

At the C-terminus of the IgA HC lies an 18 amino acid extension known as the tailpiece. While a corresponding feature is lacking in IgG and IgE, a highly similar sequence is found at the C-terminus of the HC of IgM. For both IgA and IgM, the tailpiece is crucial to the Ig’s ability to polymerise into primarily dimers and pentamers, respectively.

**Figure 2:**
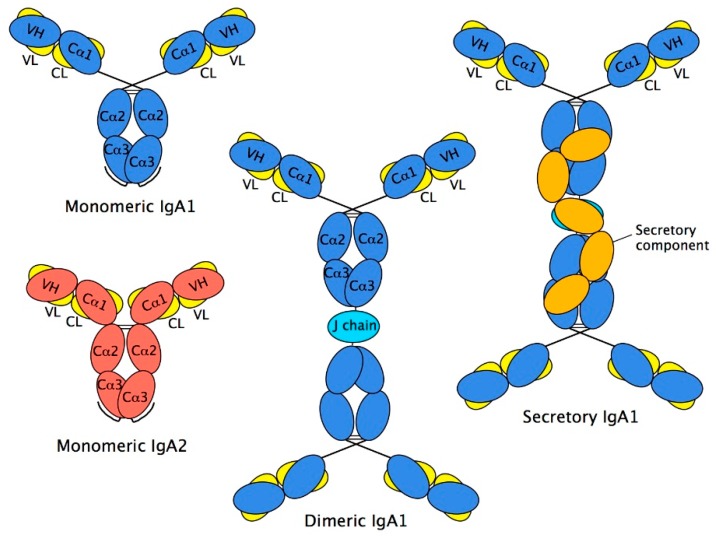
Schematic diagram of IgA structures—monomeric, dimeric, and secretory IgA. In IgA1, the heavy chain domains are in blue, and those of the light chains in yellow. In IgA2, the heavy chain domains are in red, and the light chain domains in yellow. The tailpieces are shown as extensions to the C-termini of the Cα3 domains in the monomeric forms. Dimeric and secretory forms of IgA2 are not depicted. J chain, which is present in both dimeric and secretory IgA, is shown in cyan. The domains of secretory component, derived from the extracellular region of pIgR, are present in secretory IgA and are shown in orange.

Two HCs and two LCs are organised into two Fab regions (each comprising VH, Cα1, VL, and CL domains), responsible for binding to antigen, linked via the hinge region to a single Fc region (comprising two Cα2 and two Cα3 domains), responsible for triggering elimination processes (Figure 2). The interaction between chains is stabilised by disulphide bonds between the HCs and LCs within the Fab region and between the two HCs at the Cα2 domains, and by close pairing of opposing domains: VH with VL, Cα1 with CL, and one Cα3 domain with the other one. Such pairing relies on an array of non-covalent interactions, chiefly hydrogen bonds and van der Waals contacts, between the domains involved. 

**Figure 3:**
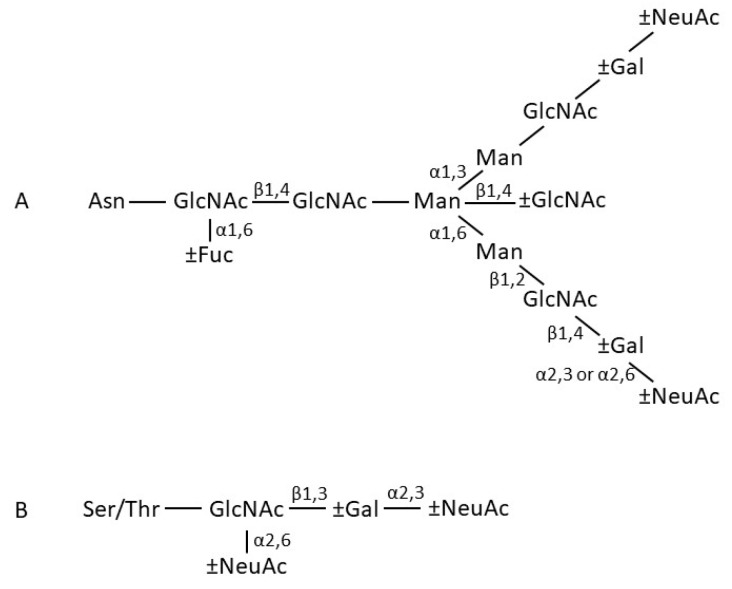
Schematic structures of IgA (**A**) *N*-linked and (**B**) *O*-linked glycan side chains. Structure (**A**) occurs in both IgA1 and IgA2, while structure (**B**) is present only attached to the hinge region of IgA1. NeuNAc, *N*-acetyl neuraminic (sialic) acid; Gal, galactose; GlcNAc, *N*-acetyl glucosamine; Man, mannose; Fuc, fucose; GalNAc, *N*-acetyl galactosamine. ±Gal, ±NeuNAc, or ±Fuc indicate that some chains terminate at the preceding sugar.

The Cα2 domains do not form a close pair, but instead have *N*-linked oligosaccharides that overlie the surfaces normally involved in pairing. *N*-linked oligosaccharides in fact make rather a significant contribution of the total mass of IgA, accounting for 6–7% of the mass of human IgA1, and 8–10% of the mass of human IgA2 [9]. The aforementioned Cα2 domain sugars are found in both IgA1 and IgA2, attached to residue Asn263. Both subclasses have another *N*-linked sugar attached to the tailpiece at residue Asn459. Recently, it has been reported that the glycans attached at Asn459 can interact directly with certain viruses and thereby neutralise them [10]. Human IgA2 has further *N*-linked sugars attached at residues Asn166 in the Cα1 domain and Asn337 in the Cα2 domain. IgA2 molecules of the IgA2m(2) allotype have a further *N*-linked sugar attached at Asn211 in the Cα1 domain. In terms of composition, the *N*-linked sugars of serum and secretory IgA comprise a family of related structures centred on a biantennary mannosyl chitobiose core, with a small proportion being more branched, mostly with triantennary structures. Fucosylation level varies, as does the numbers of sugars (galactose and sialic acid) found at the branch termini (Figure 3) [11,12,13]. Further glycosylation complexity arises through the attachment of usually between 3 and 6 core 1 and/or Tn *O*-linked sugars, composed principally of *N*-acetyl galactosamine, galactose, and sialic acid, to the hinge of IgA1 [12,13]. These *O*-linked glycans introduce further heterogeneity, since they consist of a family of structures, varying in terms of the presence or absence of sialic acid and galactose.

### 2.2. IgA Fab Region

In terms of structural components unique to IgA, within the Fab region it is the Cα1 domain that constitutes the IgA-specific component, with the VH, VL, and CL being common to other Ig classes. Solved X-ray crystal structures of the Fab regions of mouse IgA myeloma proteins have provided earlier structural insights. From two different plasmacytoma IgAs, the elbow bend angle between the VH and Cα1 domains was seen to range between 133 and 145°, suggesting a degree of flexibility within the Fab region [14,15]. However, more recently, the crystal structure of a human IgA1 Fab has been determined at high resolution [16]. The position of the disulphide between the LC and HC, together with the markedly hydrophobic interface between the VH and Cα1 domains, appears to constrain the IgA1 Fab, making it somewhat rigid. When compared to a matched IgG featuring the same VH and VL domains, the IgA1 Fab exhibited a difference of about 5° in the elbow angle from that in IgG. It has been suggested that the greater rigidity inherent in IgA1 Fab may exert subtle allosteric effects on the antigen binding site with resultant impact on antigen binding affinity. Such considerations are relevant to engineering of therapeutic antibodies, and are explored in depth elsewhere [17].

The IgA subclasses differ in the arrangement of their interchain disulphides, including those between LC and HC within the Fab region. While IgA1 and IgA2(m)2 have the usual disulphide bridges between HC and LC, these are located at different positions—between a common Cys in LC and Cys133 in IgA1 HC and Cys220 in IgA2m(2) HC. These HC Cys are located close to the VH–Cα1 interdomain region and at the C-terminal end of the Cα1 domain (penultimate residue), respectively. Remarkably, in IgA2m(1), such HC–LC disulphides are generally lacking. Instead, disulphide bridge links the two LCs, and the association between HC and LC is stabilised by non-covalent interactions.

### 2.3. IgA Fc Region

**Figure 4:**
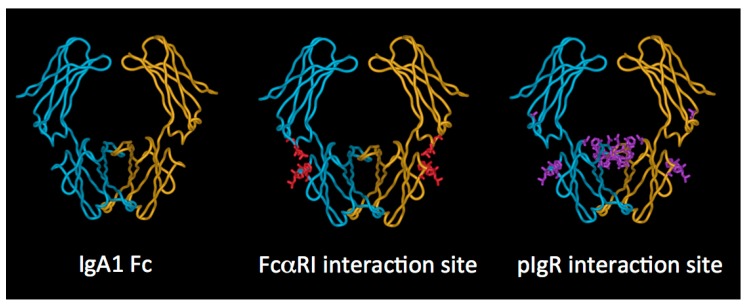
X-ray crystal structure of human IgA1 Fc generated from PDB accession code 1OW0 using only the IgA coordinates. One heavy chain is shown in blue, the other in gold. Residues critical for binding to FcαRI are shown in red on the middle image, and those implicated in the interaction with pIgR are shown in purple on the right hand image.

Turning to the Fc region, important structural information has been gained from the solved X-ray crystal structures of human IgA1 Fc in complex with the extracellular domains of FcαRI [18] and with the staphylococcal protein SSL7 (Figure 4) [19]. In terms of overall configuration, the structure of the Fc region is similar to that of IgG and IgE, but there are important distinctions. Notably, the location of the disulphide bridges between the two HCs, and the attachment sites and positions of the *N*-linked glycans are different in IgA from these other Ig classes. 

Unlike IgG, where there are numerous inter-HC disulphide bridges in the hinge region, IgA lacks hinge disulphides and, instead, has disulphide bridges between the upper reaches of the Cα1 domain (Figure 2). Thus, Cys242 in each HC can link to Cys299 in the opposite HC. Further disulphide bonds are presumed to exist, for example, between Cys241 in each HC, or between Cys299 in each HC, or between Cys241 in one HC and Cys301 in the other, but the truncated forms of IgA1 Fc used in crystallisation did not allow direct resolution of these.

The Cα2 domains are not closely paired, a feature similar to the equivalent domains in IgG (Cγ2) and IgE (Cε3). Such non-pairing might be expected to expose a considerable area of domain surface to solvent, but this potentially less stable scenario is avoided to some extent due to attachment of *N*-linked glycans at Asn263. The sugar moieties attached at this site lie over the outer surfaces of the Cα2 domains and, in doing so, bury around 930Å^2^ per Fc from solvent contact. The glycans also make contact with the Cα3 domains, thereby burying another 914Å^2^ per Fc from solvent, further stabilising the Fc region.

The 18 amino acid tailpiece at the C-terminus of each HC was missing from the IgA1 Fc fragments used for crystallisation, and hence no information on its structure was obtained. Recently it has been modelled to occupy a range of conformations [20]. The tailpiece carries a cysteine residue at position 471, and the potential linkages that this cysteine residue may make with other “free” Cys residues in IgA remains somewhat of an enigma.

### 2.4. Structure of Monomeric IgA

As with other Igs, the inherent flexibility of intact monomers of IgA tend to frustrate crystallisation efforts. Thus, in order to probe the conformation of entire IgA monomers rather than the separate Fab and Fc regions, lower resolution techniques, including electron microscopy (EM), and more recently, X-ray and neutron scattering of IgA in solution, have been used. These have been useful in predicting the overall dimensions of IgA molecules, and have led to an understanding that the IgA1 has a greater average Fab centre to Fab centre distance than IgA2: 16.9 nm for IgA1 compared with just 8.2 nm for IgA2 [21,22,23,24,25,26]. 

**Figure 5:**
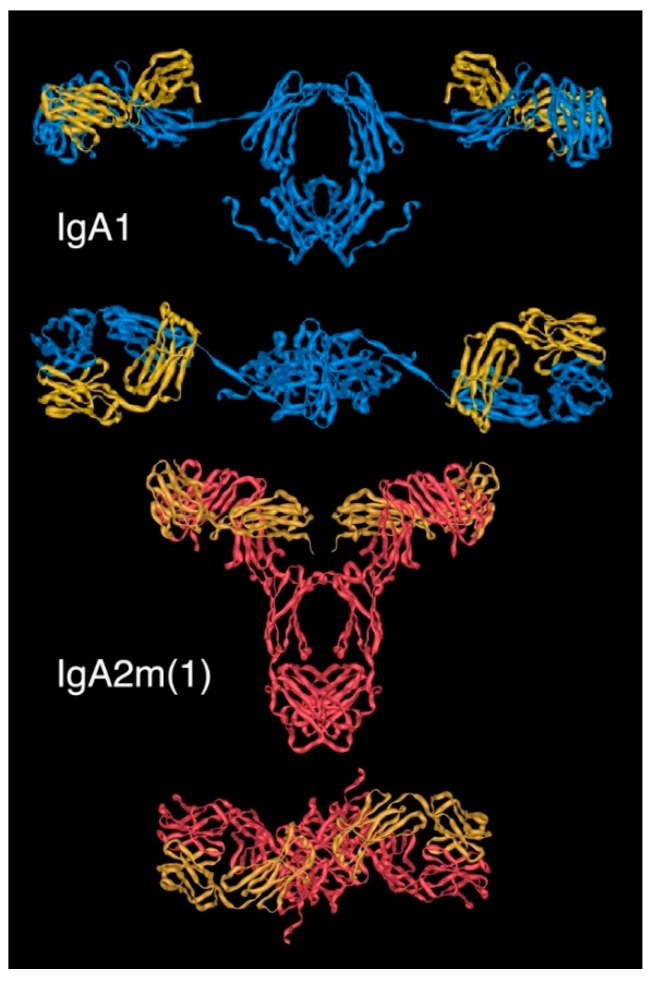
Molecular models of human IgA1 and IgA2(m)1 using coordinates from PDB accession codes 1IGA and 1R70, respectively, seen face on (upper image in each case) and from above (lower image in each case). In IgA1, heavy chains (HCs) are shown in blue and light chains (LCs) in yellow, while in IgA2m(1), HCs are shown in red and LCs in yellow.

Models arising from solution scattering studies originally suggested that both human IgA subclasses adopt average T-shaped structures (Figure 5), which presumably reflected averages of the different conformations available to these molecules as a result of flexibility. Indeed, more recent work using these techniques has reported IgA1 to have an extended Y-shaped structure, with the Fab regions positioned well away from the Fc, in keeping with previous electron micrographs. Given the major advances made in recent years in cryogenic electron microscopy (cryo-EM), it can be envisaged that definitive understanding of the structure of monomeric IgA is likely to emerge from this technique. 

### 2.5. Dimeric IgA

The IgA destined for the mucosal surfaces is produced locally to the mucosa in polymeric form. These are principally dimers comprising two IgA monomers covalently linked to an additional polypeptide known as joining chain or J chain. J chain is a 15 kDa polypeptide, expressed by antibody-producing cells, and is also present in larger IgA polymers and pentameric IgM. It is incorporated into polymeric IgA or IgM prior to secretion [27]. In the case of IgA, marginal zone B and B-1 cell-specific protein (MZB1) has been shown to promote J chain binding to IgA in plasma cells [28]. J chain is very highly conserved across species (mammals, birds, reptiles, fishes, and amphibian) and is not known to resemble any other protein. It has one *N*-linked glycan attached at Asn48 which exists in five major forms, principally sialylated biantennary complex structures [13]. J chain’s ability to join HCs in polymeric Igs relies on two key Cys residues, from amongst the eight cysteines it possesses. Six of the eight are involved in interchain disulphide bridges (Cys12–Cys100, Cys17–Cys91, and Cys108–Cys133) [29,30]. Presently, the three-dimensional structure of J chain is unresolved. Models have tended to favour a two-domain structure [30,31].

Early studies of dimeric IgA structure utilised EM to view myeloma IgA preparations. It was seen to have a double-Y shape, in which the Fc regions joined to each other via their C-terminal regions. The length of the joined Fc region was in the range 125–155 Å, consistent with two Fc regions of about 65 Å long being arranged end-to-end (Figure 2). The J chain is interposed between the two Fc regions, and links to each of the monomers through disulphide bridges formed between the penultimate Cys residues of the tailpieces (Cys471) and the two J chain cysteines alluded to above (Cys14 and Cys68). The critical roles played by these cysteines in the linkage has been verified through targeted mutagenesis of both the tailpiece and J chain [32,33]. In keeping with these observations, solution structure analysis of dimeric IgA1 have predicted a near-planar structure with end-to-end Fc contacts, although in this study, the J chain structure and orientation used in the modelling was arbitrary [34]. Further analysis, possibly from techniques such as cryo-EM, will be necessary to provide an in-depth view of the relative arrangement of Fc regions and J chain.

### 2.6. Secretory IgA

In external secretions, the predominant form of IgA is SIgA, which derives from local synthesis by Ig-producing cells in organised mucosal-associated lymphoid tissues, most of which are committed to the IgA isotype. SIgA is mostly in dimeric form, with some tetramers also being present. The relative proportions of each varies from mucosal site or secretion. For example, in saliva and milk, the ratio of dimeric/tetrameric SIgA is around 3:2. Secretions can also contain some monomeric IgA, but again, the amounts vary. In saliva and milk, about 5–10% of the IgA is monomeric, whereas in cervical fluid, a much higher proportion can be present [35].

**Figure 6:**
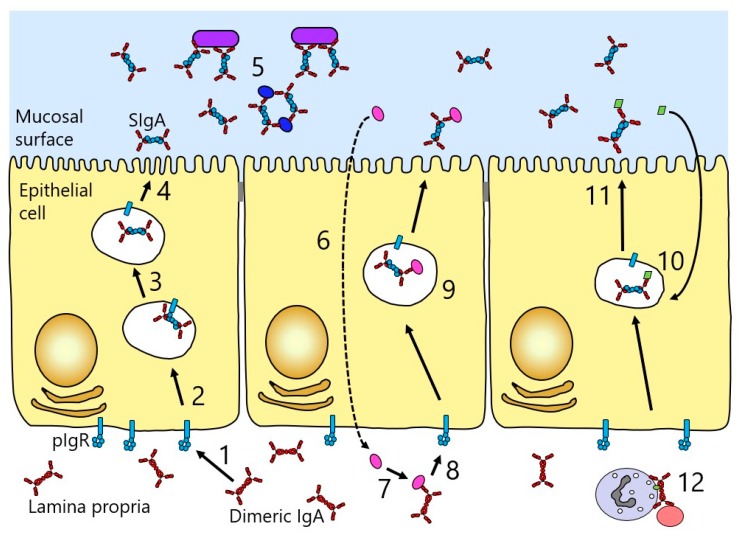
Schematic diagram illustrating the role of pIgR in transporting IgA across the mucosal epithelium. Gut epithelium is shown as an example. (**1**) Dimeric IgA (shown in red) produced locally at the mucosal surface binds pIgR (cyan) at the basolateral surface of the epithelial cell layer. (**2**) The complex is internalised and undergoes vesicular transport across the cell. (**3**) pIgR is cleaved to release secretory component (SC), which becomes disulphide-bonded to the dimeric IgA. (**4**) At the apical surface, SIgA is released. (**5**) SIgA binds to and neutralises bacterial and viral pathogens (shown in purple and dark blue). (**6**) Some pathogens (shown in bright pink) may gain access to the lamina propria underlying the epithelium. (**7**) Such pathogens can be bound by dimeric IgA. (**8**) The dimeric IgA–pathogen complex binds to pIgR. (**9**) The pathogen is carried out across the epithelium and released back out into the lumen. (**10**) Some pathogens (shown in lime green) can be intersected by dimeric IgA during transit across the epithelial cells. (**11**) The pathogen is ejected upon release of SIgA at the mucosal surface. (**12**) Dimeric IgA can mediate clearance mechanisms against pathogens (in salmon pink) through engaging phagocytes.

Another factor accounting for the high relative concentration of IgA in secretions is the presence of a receptor known as the polymeric Ig receptor (pIgR), which mediates the specific transport of polymeric Igs across the mucosal epithelium into the secretions (Figure 6). pIgR is expressed on the basolateral surface of epithelial cells lining mucosal sites, and binds and transports only polymeric Igs. At mucosal surfaces, the predominant ligand is dimeric IgA, since the larger size of IgM restricts diffusion from serum, and hence, the smaller, and locally-produced, dimeric IgA is preferentially transferred [36].

pIgR is a single polypeptide receptor, comprising a ~620 amino acid extracellular portion which folds up into five Ig-like domains with particular homology to Ig variable domains, a 23 amino acid transmembrane section, and an internal tail of around 103 amino acids [37]. The extracellular domains, named D1–D5 from the *N*-terminus, are each stabilised by one or more internal disulphide bridges, and are decorated by seven *N*-linked glycans. Between the end of D5 and the membrane lies a short stretch of non-Ig-like sequence.

Transport of dimeric IgA across the epithelium (transcytosis) involves its binding to pIgR at the basolateral surface of the epithelial cell, followed by internalisation and transport via vesicular compartments to the apical surface of the cell (Figure 6). During the process, pIgR is cleaved between D5 and the membrane to release a major fragment of the receptor referred to as secretory component (SC). A disulphide bridge forms between SC and dimeric IgA, and when the complex is released at the apical surface, SC remains as part of the released IgA, then known as SIgA. EM studies of SIgA from colostrum show a double Y-shaped configuration.

Domains D1–D3 of pIgR are known to play critical roles in binding to dimeric IgA, with domains D4 and D5 also making smaller contributions. In particular, loops lying at the end of D1, akin to the complementarity determining regions (CDR) of variable domains, are central to the binding and are known to lie close to each other based on the solved X-ray crystal structure of the domain [38,39,40]. Residues in CDR1, CDR2, and CDR3 have been implicated in the binding to dimeric IgA [37].

Turning to the elements of dimeric IgA involved in the interaction, it is believed that the initial interaction involves engagement of D1 of pIgR with an exposed loop (residues 402–410) and other close lying residues (Phe411, Val413, Thr414, Lys377) on the Cα3 domain of IgA, along with a region on the Cα2 domain (Pro440–Phe443) lying at the Cα2–Cα3 domain interface (Figure 4) [41,42,43]. Thereafter, a disulphide bound formed between one of two cysteine residues in D5 of pIgR (Cys468 or Cys502) and Cys311 in the Cα2 domain of IgA anchors SC and dimeric IgA together [44]. It has also been demonstrated that direct interactions between J chain and pIgR occur [45].

**Figure 7:**
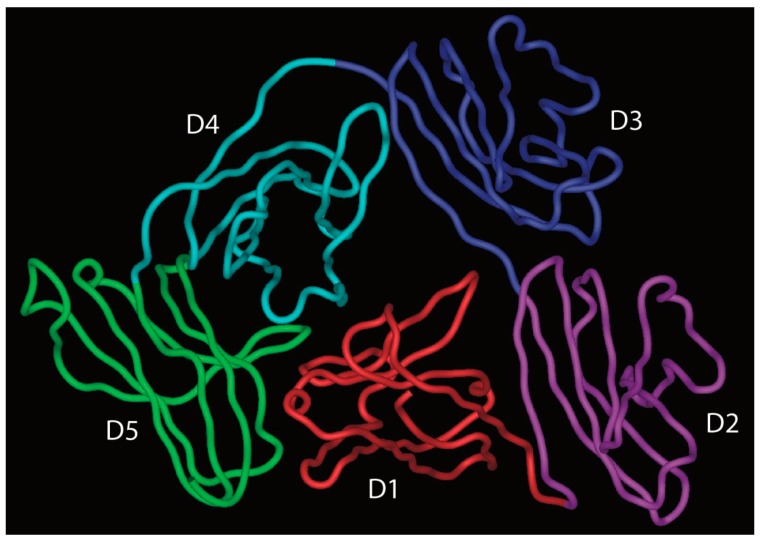
Crystal structure of the extracellular domains of human pIgR (using coordinates from PBD accession code 5D4K). Each of the five domains (D1–D5) has been coloured differently.

More recently, the structure of free SC has been elucidated by X-ray crystallography and shown to adopt a triangular arrangement, with a large interface between domains D1, D4, and D5, which buries some 1480 Å^2^ of surface area from solvent contact (Figure 7) [46]. The five domains lie in a plane, giving the triangle shape a thickness similar to that of a single domain (about 40 Å). To further explore SC structure and its relationship to function, the same study used double electron–electron resonance spectroscopy on spin-labelled variants of SC in solution as a means to explore the flexibility of the protein domains. This analysis confirmed the crystal structure to represent the predominant solution structure of free SC at the D1–D5 interface. However, when the spin-labelled SC was incubated with dimeric IgA, a dramatic separation of D1 and D5 was apparent, consistent with an increase in distance of more than 42Å between these domains, resulting in a final separation of more than 85 Å. Analysis of the binding characteristics of shortened constructs of SC supported the key role of D1 in binding to dimeric IgA and indicated a role for D5 in mediating non-covalent interactions with dimeric IgA [46]. The results also suggest that D2, and possibly D3, contribute to binding either directly or through promoting interactions between D5 and dimeric IgA. Thus, we are left with a current model that involves opening up of the pIgR extracellular structure upon binding to SIgA, with initial contact through D1, but later involvement of the other pIgR domains. The final separation of D1 and D5 would be sufficient to allow engagement of D1 and D5 with domains in the same IgA monomer or across the two different IgA monomers present in the dimer. 

## 3. IgA Function

### 3.1. Neutralisation

Through direct engagement of their antigen binding sites with antigens on pathogens, IgA molecules neutralise or block the activity of a range of viruses, bacteria, and protozoa, and prevent their attachment to host cells [47]. Similarly, binding of IgA to pathogenic products such as toxins can neutralise their activity and prevent the disease symptoms associated with them [48]. The attachment of several types of pathogenic microorganisms to the mucosal surfaces can be prevented by the interaction of the glycans on IgA with sugar-dependent receptors or fimbriae on their surfaces [10,49,50,51]. Thus, IgA contributes to immune exclusion, a process by which the adsorption of pathogens to mucosal surfaces is prevented through agglutination, such that the aggregates formed are unable to penetrate though the mucus that lines mucosal surfaces. The multiple antigen binding sites of SIgA enable both high avidity binding and crosslinking of particulate matter, resulting in efficient blocking activity. Moreover, IgA can interact with other innate defence factors in mucosal secretions to enhance immune protection. These include mucins [52,53], lactoferrin, and the lactoperoxidase system [54]. 

In vitro studies suggest that mucosal IgA can also mediate protective functions during its passage through the epithelium or by carrying pathogens or their products encountered on the basolateral side of the epithelium out across the epithelium (Figure 6) [55]. The latter reflects the fact that pIgR can transport dimeric IgA alone or in complex with antigen. This mechanism can drive removal or excretion of soluble antigens from various origins, as well as viral particles [56]. Antigen-specific dimeric IgA has been seen in vitro to neutralise endocytosed bacterial lipopolysaccharide (LPS) within epithelial cells, whilst undergoing pIgR-mediated transcytosis. Following colocalisation within the apical recycling compartment, the IgA was able to prevent the proinflammatory events usually triggered by LPS [57]. Similarly, while undergoing epithelium transcytosis, dimeric IgA targeted to certain viruses have been able to block viral growth, seemingly following intersection of the IgA and viral proteins in the apical recycling endosomes. Such effects have been reported for Sendai virus [58], influenza virus [59], measles virus [60], rotavirus [61,62], and HIV [63,64]. However, questions remain as to whether these processes reflect the situation in vivo, although experiments in mouse models suggest that there may be some physiological relevance [65,66]. 

### 3.2. Complement Activation

IgA lacks the site for C1q binding present in IgG and does not bind C1q, and therefore is not expected to activate the classical pathway of complement. Interestingly, a recent study looking at complement-dependent cytotoxicity of B cells by CD20-specific IgA suggested that complement was activated by IgA. However, in vivo, the activity of the anti-CD20 IgA to deplete B cell targets was not abrogated in C1q- or C3-deficient mice, suggesting that complement activation was not the predominant killing mechanism in action [67]. The ability of IgA to activate the alternative pathway of complement has been somewhat contentious, but the prevailing view is that the reported activation is likely via the lectin pathway as a result of binding to mannose-binding lectin [68]. However, the ability to activate via this route is likely dependent on glycosylation status.

### 3.3. Interaction of the IgA Fc Region with Host Receptors

In addition to the above-mentioned functions, IgA mediates a variety of effector functions through interaction with a number of different host receptors expressed on various cell types. The interaction with pIgR and the resultant transport into mucosal secretions has already been discussed. Now, we will turn to consideration of the IgA-specific receptor FcαRI, a key means by which IgA can trigger clearance mechanisms against invading pathogens. Other receptors which have been described to have specificity for IgA are generally less well characterised in terms of their roles and will not be addressed further here. These include Fcα/µR, which exhibits specificity for polymeric forms of IgA and IgM, in the case of IgA through a site at the Cα2–Cα3 domain interface [69]; transferrin receptor (CD71), which has been implicated in retrograde transfer of SIgA immune complexes back through the epithelium [70]; a microfold (M) cell receptor, possibly Dectin-1, which may mediate reverse transcytosis of SIgA immune complexes through M cells [71]; dendritic cell (DC)-specific intercellular adhesion molecule-3-grabbing non-integrin (DC-SIGN), which appears to take up SIgA immune complexes into sub-epithelial dendritic cells [72]; the inhibitory IgA receptor Fc receptor-like 4 (FcRL4) thought likely to be important for immune complex-dependent regulation of B cells [73]; the asialoglycoprotein receptor (ASGPR) on hepatocytes, which mediates clearance of IgA from the circulation [74]; β-1,4-galactosyltransferase 1, which, along with CD71, has been identified as a potential IgA receptor on kidney mesangial cells [75]; and lastly, the putative receptor for SC and SIgA on eosinophils [76].

### 3.4. FcαRI

Although a less closely related member, FcαRI belongs to the Ig Fc receptor family, which also features specific receptors for IgG (FcγRI, FcγRII and FcγRIII) and IgE (FcεRI) [77,78,79]. It is expressed on neutrophils, eosinophils, monocytes, macrophages, Kupffer cells, and some DC subsets. Also known as CD89, it is encoded by a gene lying on chromosome 19, within the leukocyte receptor cluster (LRC) close to killer cell immunoglobulin-like receptors (KIR) and leukocyte immunoglobulin-like receptors (LILR) receptors. In contrast, other Fc receptors in the family are clustered on chromosome 1. In keeping with this gene location, FcαRI shares closer amino acid similarity with LRC members than with the IgG and IgE Fc receptors. 

FcαRI is organised into two extracellular Ig-like domains, a transmembrane segment, and a short cytoplasmic tail devoid of signalling motifs. It associates with a dimer of the FcR γ chain, a short transmembrane polypeptide originally characterised as a component of the IgE receptor, FcεRI. The γ chain carries two immunoreceptor tyrosine-based activation (ITAM) motifs within its cytoplasmic region, important for signalling to the cell interior upon receptor crosslinking by binding to IgA-containing immune complexes or to IgA concentrated on a pathogen surface. The outcome of such signalling can be a range of responses depending on the cell involved, from phagocytosis, superoxide generation (respiratory burst), release of cytokines, chemoattractants, or inflammatory mediators, through to release of neutrophil extracellular traps (NET) [80,81]. On the other hand, binding of monomeric IgA to FcαRI has been reported to trigger inhibitory signals via the γ chain ITAM as opposed to the aforementioned activatory ones. Such inhibitory ITAM (ITAMi) signalling is considered to dampen down excessive IgA immune complex-mediated responses. The underlying signalling processes and the specifics of responses are detailed elsewhere [82].

Alternatively spliced isoforms of FcαRI exist, with those known as a.1 and a.2 being expressed on phagocytes [83,84]. The a.1 version has a molecular weight of 55–75 kDa on neutrophils and monocytes, while additional glycosylation renders it a little heavier (70–100 kDa) on eosinophils. The a.2 version is lacking 22 amino acids from the second extracellular domain, and is only present on alveolar macrophages. In terms of allelic variation, a common, nonsynonymous, single nucleotide polymorphism (SNP) has been described in the coding region of FcαRI, which results in a change of residue 248 from Ser to Gly within the cytoplasmic domain [85].

The structure of the ectodomains of FcαRI has been solved at high resolution, in complex with the Fc region of IgA1 [18]. The globular extracellular domains lie at an angle of around 90° to each other, and it is notable that their relative orientation is very different from the corresponding domains of other Fc receptors [18,86].

FcαRI binds both subclasses of human IgA with similar affinity, and also engages both serum IgA (monomeric) and SIgA (polymeric), albeit with some differences in outcome [82]. However, it has been observed on polymorphonuclear leukocytes that SIgA cannot bind to FcαRI in the absence of CR3 or Mac-1 [87]. The affinity of FcαRI for IgA molecules in solution is low (K_a_ of approximately 10^−6^ M^−1^), but IgA immune complexes, or IgA aggregated for example on a pathogen surface, bind with higher avidity. The crystal structure of the complex of the ectodomains of FcαRI and IgA1 Fc revealed that each IgA Fc region is capable of binding two FcαRI molecules [18]. The physiological relevance of this observed stoichiometry is a subject of some conjecture. The site of interaction on IgA, originally defined by mutagenesis [88,89,90] and further defined by crystallography [18], lies at the Fc domain interface, with important contributions from Cα2 residues Leu257 and Leu258 and Cα3 residues Met433, Leu441, Ala442, Phe443, and the aliphatic portion of Arg382 (Figure 4). On the receptor, the hydrophobic core of the interaction relies on contributions from a region in the membrane distal domain (Tyr53, Leu54, Phe56, Gly84, His85) with contributions also from Lys55 [18,91,92]. This mode of Fc receptor–Ig interaction is very different from the FcγR–IgG and FcεRI–IgE interactions, which involve sites on the upper reaches of the respective Fc regions, and on the membrane proximal domains of the respective receptors [86].

The contribution of *N*-linked glycans, both on FcαRI and IgA, in the interaction have been investigated. Studies using a glycoengineering approach to generate IgAs carrying distinct homogeneous *N*-glycans have indicated that different glycoforms of IgA1 and IgA2 do not exhibit radically different binding to FcαRI [20], in keeping with earlier analysis that showed that variation in or lack of the *N*-linked glycans at Asn263 in the Cα2 domain did not significantly impact on binding to FcαRI [12,93]. In contrast, specific *N*-linked sugar moieties on FcαRI have been shown to impact on binding to IgA [20,94]. A FcαRI glycovariant with oligomannosidic *N*-glycans has been reported to bind IgA 2–3 times more tightly than variants with complex *N*-glycans [20], while deglycosylation of FcαRI at Asn58 has been shown to increase binding to IgA [94].

Recently, binding of FcαRI to IgA has been demonstrated to propagate conformational changes within IgA as far as the hinge region [95]. Thus, FcαRI binding was shown not only to cause a decrease in IgA Fc intradomain and interdomain flexibility, but also to impact on the hinge, such that binding of lectins to the IgA1 hinge was affected.

It has been reported that peptide mimetics, consisting of either linear or cyclised peptides of 7–18 amino acids spanning regions of FcαRI or IgA known to be involved in the interaction site, may serve as a means to inhibit IgA–FcαRI interactions [96]. Such peptides were shown to reduce IgA effector functions mediated through FcαRI such as phagocytosis and production of activated oxygen species. Blocking strategies based on peptides such as these, or on antibodies directed against FcαRI, have been proposed as possible routes to prevent undesirable inflammatory conditions triggered through aberrant IgA immune complexes [79,97].

Specific elements of the innate immune system are also known to interact directly with FcαRI and impact on IgA binding. Thus, pentraxins such as C reactive protein and serum amyloid P component, which adopt pentameric ring-like structures, have been shown to bind to FcαRI, in part, via a similar region as IgA. Although the pentraxin interaction site on FcαRI appears to be more extensive than that responsible for binding IgA, these acute phase proteins are able to competitively inhibit IgA binding [98].

## 4. Circumvention of IgA Function by Pathogens

On the basis of phylogenetic and diversity analysis, the IgA–FcαRI interaction has been proposed to be the focus of an evolutionary arms race between pathogens and humans [99,100]. The site on IgA central to the interaction, which has been conserved in order to bind FcαRI, has been placed under pressure to evolve by IgA binding proteins that certain pathogens produce. These IgA binding proteins have evolved to interact with the same site, thereby subverting the IgA response, and driving an iterative selective process in which both mammalian and pathogen proteins have continued to evolve in an attempt to “outsmart” the other. In fact, targeting of the FcαRI interaction site is just one of the strategies that pathogenic microorganisms have used to circumvent the protective capabilities of IgA. The existence of different IgA-targeting mechanisms, together with the fact that these mechanisms seem to have arisen independently in different organisms, suggests that they offer significant benefits to microorganisms by allowing easier mucosal colonisation and spread. Examples include the IgA binding proteins mentioned above and the production of enzymes that cleave and inactivate IgA, which will be discussed in more detail below, and the generation of proteins that bind SC or pIgR and aid adherence and invasion within the mucosae [101,102,103,104].

### 4.1. Bacterial IgA Binding Proteins

Certain important pathogenic bacteria, including Group A and B streptococci and *Staphylococcus aureus*, express proteins on their surface, which bind specifically to IgA. Group A streptococci, which cause a range of diseases from mild skin and throat infections to life-threatening systemic conditions, express Sir22 and Arp4, while group B streptococci, responsible for serious, sometimes deadly, infections in new-born infants, express the unrelated β protein [105,106,107]. *Staphylococcus aureus*, which can cause bacteraemia, infective endocarditis, and skin and soft tissue infections, expresses an IgA binding protein known as Staphylococcal superantigen-like protein 7 (SSL7). Despite these proteins not being related to each other, all bind at the Cα2–Cα3 interdomain region of IgA Fc at sites that overlap with that for FcαRI [19,108,109]. They have been shown to competitively inhibit FcαRI binding; further, the streptococcal proteins have been demonstrated to block triggering of elimination mechanisms via FcαRI. Thus, these IgA binding proteins provide the bacteria in question with effective ways to evade IgA-mediated clearance.

### 4.2. Bacterial Proteases That Target IgA

The protective capabilities of IgA can also be compromised through the actions of proteolytic enzymes produced by a number of important pathogenic bacteria. These proteases all cleave in the hinge region of IgA. With few exceptions, they act specifically on the extended hinge region of IgA1, and do not cleave IgA2. Such IgA1 proteases are produced by bacteria responsible for infections of the oral cavity, such as *Streptococcus sanguis*, *Streptococcus mitis*, and *Streptococcus oralis,* and of the genital tract, such as *Neisseria gonorrhoeae*, suggesting that they afford an advantage to the bacteria in gaining a foothold at mucosal surfaces. In addition, they are produced by bacteria responsible for meningitis (*Haemophilus influenza*, *Neisseria meningitidis*, and *Streptococcus pneumoniae*). 

The IgA1 proteases appear to have evolved several times over since those from different bacterial species tend not to share common features. Indeed, they represent a range of protease types, with some being metalloproteases, others being serine proteases, and yet others being cysteine proteases [110]. By separating the antigen-binding region of IgA from the Fc region critical for binding to host FcαRI, IgA1 proteases perturb normal IgA-mediated protection mechanisms and leave the bacteria free to proliferate [111]. 

**Figure 8:**
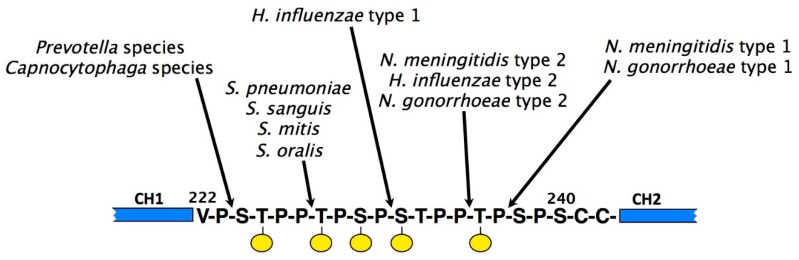
Amino acid sequence in the hinge region of human IgA1 and the cleavage sites of various IgA1 proteases. The IgA1 hinge contains a duplicated octapeptide sequence that is missing in IgA2. *O*-linked glycans are represented by yellow circles.

Each IgA1 protease cleaves a specific site within the IgA1 hinge, either a Pro–Thr or a Pro–Ser peptide bond (Figure 8). In order for IgA1 proteases to recognise the IgA1 hinge as a substrate, it has become clear that not only sequence elements within the hinge itself are important [112,113], but, at least for some IgA1 proteases, also specific regions of the IgA1 protein lying well beyond the hinge. Thus, for efficient cleavage to occur, the susceptible bond is required to be positioned at a suitable position relative to the Fc [114], and some proteases also require the presence of elements within the Fc region of IgA1 [115,116]. Specifically, Cα3 domain residues Pro440–Phe443, which as mentioned above form part of the interaction sites for FcαRI and pIgR, have been shown to be a requirement for cleavage of IgA1 by the *N. meningitidis* type 2 IgA1 protease, while for the *H. influenzae* type 2 enzyme, different Cα3 residues predicted to be involved in pIgR interaction are required for cleavage to proceed [116]. Echoing the case with IgA binding proteins, these requirements suggest that IgA1 proteases may have commandeered conserved host receptor sites for their own benefit. One can envisage an interaction between IgA1 protease and the IgA1 molecule as a whole, with the protease engaging with elements within the Fc region as a means to stabilise a particular IgA conformation and aid positioning of its active site next to the IgA1 hinge. Indeed, the solved X-ray crystal structure of an *H. influenzae* IgA1 protease is consistent with such a possibility [117].

A more detailed understanding of the molecular basis of IgA1 hinge cleavage by IgA1 proteases may have therapeutic application. For example, following earlier work to identify possible inhibitors for IgA1 protease [118,119], small molecule non-peptidic inhibitors for *H. influenzae* IgA1 protease have recently been described in the first steps towards development of potential therapeutics for antibiotic-resistant *H. influenzae* strains [120]. Further, it has been proposed that IgA1 proteases may have utility as therapeutic options to degrade pathogenic immune complexes of aberrantly glycosylated IgA1 in IgA nephropathy, a common cause of kidney disease [121,122].

## 5. IgA Developability

Specific IgA is often found elevated in the serum and/or secretions after immunisation. While vaccination via the systemic route tends to generate serum responses, vaccination through the intranasal or oral route can elicit protective mucosal responses [123]. As a prime example, oral cholera vaccination is well established as a means to induce protective mucosal IgA responses [124]. As another example, studies in mice have shown that a nasal vaccine is sufficient to prevent *Streptococcus pneumonia* colonisation, registering high levels of IgA and IgG in plasma and nasal washes. However, this protective action was abrogated in IgA deficient mice [125]. In the context of viruses, neutralising IgA antibodies against HIV can be found in the serum of survivors or vaccinated HIV patients [126,127], and serum and salivary IgA against polio virus can be found elevated upon vaccination with live attenuated viruses [128]. In mice, immunisation against reovirus has been demonstrated to lead to an increase of serum and gut IgA, which proved to be essential to prevent reovirus infection [129]. A similar outcome was observed in mice immunised with influenza virus hemagglutinin, where the induced IgA response provided protection against influenza infection [130]. 

The above studies present a snapshot of the protective role that IgA can play against bacterial or viral infections, both in serum and mucosal secretions. Since specific IgA can clearly be beneficial in clearing viral or bacterial infections, passive administration of IgA is an attractive option in cases where the immune response is comprised or where insufficient time, or other logistical hurdles, prevent generation of a timely and robust response through active immunisation. Moreover, with regard to the protection of mucosal sites, effective vaccination requires the correct antigen, adjuvant, and delivery route to promote a robust and protective response. Hence, the use of passive immunisation, by direct delivery of specific antibodies, can present an alternative for the protection of mucosal surfaces. However, it remains challenging to create a delivery route, especially for the gut mucosa. 

### 5.1. Advantages of IgA-Based Therapeutics

The therapeutic antibody field is currently dominated by IgG-based mAbs. The advantages of opening up this arena to include IgA-based mAbs are becoming increasingly apparent, piquing interest in both academia and industry [79,131,132,133]. One advantage is the new prospects it offers in terms of intellectual property, in what is already a complex landscape [134]. Secondly, as will be explored further below, IgA mAbs are known to be highly effective at recruiting immune cells, and neutrophils in particular, to deliver potent killing mechanisms, making the IgA–FcαRI axis an important target in control of various cancers and infections. Such neutrophil-mediated tumour cell killing is considered especially important for apoptosis-resistant cells [131]. Thirdly, IgA is likely to represent the most suitable option for mucosal applications, given its prevalence and functional capabilities at such sites. Fourthly, the structural distinctiveness of IgA, especially IgA1 with its ability to bridge greater distances between antigens, may offer enhanced avidity in some scenarios. Fifthly, IgA can naturally polymerise into forms with enhanced agglutination capabilities, and which can be transported by pIgR into mucosal secretions. Finally, it is possible to use components of IgA or IgA heavy chains in combination with those of other Igs such as IgG, to explore new therapeutic possibilities.

### 5.2. Constraints of Using IgA Therapeutically and Efforts to Resolve These

Despite the numerous advantages that may be associated with the development of IgA in the therapeutic setting, there are a number of constraints or limitations that need to be addressed. For example, both pro-inflammatory and anti-inflammatory functions of IgA mediated through FcαRI have been flagged up as being of relevance to the therapeutic potential of IgA [131,135]. As a result, it will be important to establish the mechanism(s) at play in any particular treatment setting.

Another constraint is that IgA has a shorter half-life than IgG, estimated to be 4–6 days [136,137]. IgA cannot bind to the neonatal Fc receptor, FcRn, while engagement of IgG with this receptor results in a half-life of about 21 days (although it varies with subclass). The short half-life of IgA would necessitate much more frequent dosing if this class was to be used therapeutically. For example, in mouse tumour models, it has been found to be necessary to give daily injections of IgA antibodies to reach effective circulating concentrations [138]. Unless modified, use of IgA is therefore likely to be expensive and less convenient for recipients because of the frequency of dosing. This shorter half-life is in part due to clearance mediated by the ASGPR, which recognises terminal galactose residues on the glycans of IgA. Efforts have been made to extend half-life by removing *N*-linked glycosylation sites [139], generating IgA with higher terminal sialylation of *N*-glycans [140], by attaching an albumin-binding domain to either the LC or HC in order to facilitate binding to the neonatal Fc receptor FcRn [141], or by engineering in FcRn binding by generating an IgG–IgA Fc fusion [133].

A further constraint relates to efficiency issues in the expression, production, and purification of recombinant IgA mAbs of a suitably homogeneous nature. It has long been recognised that IgA production suffers from low expression levels and heterogeneous glycosylation. Systems enabling increased expression of IgA have been developed [140,142,143], and advances in general expression systems for other Igs are likely also to bring benefits [144,145]. There is interest in using plant-based systems to express IgA [146,147,148], but the implications for glycosylation must be borne in mind, especially since it is known that IgA glycosylation is impacted by expression system [149,150].

The logistics of working with IgA has been challenging due to the limited options for specifically purifying this Ab class. Jacalin, a lectin that binds to the *O*-linked sugars on the IgA1 hinge, and light chain binding protein-based strategies offer rather limited possibilities. Immobilised bacterial IgA binding proteins, or peptides derived from them, represent a feasible solution [151,152], and IgA-binding peptides selected from random peptide libraries may also have applicability in IgA purification [153].

The susceptibility of IgA1 to cleavage by IgA1 proteases may be another potential constraint to its use. However, as discussed above, mutagenesis analysis has demonstrated how this might be overcome either by engineering of the hinge itself or of the Fc region [116].

Another area for consideration in the design of therapeutic IgA mAbs are the routes to ensure complete assembly. For instance, the disulphide bridge complexity in IgA2 presents challenges [154]. The production of polymeric forms of IgA or SIgA is particularly complex, given the requirement to co-express LC, HC, and J chain, and ensure attachment of SC. However, systems to achieve this have been explored and continue to be refined [133,155,156].

A final constraint to the development of therapeutic IgA mAbs stems from the lack of suitable animal models. Since IgA1 equivalents are only found in humans and closely related apes, the use of the species normally used in experimental research (mouse, rat, rabbit) will most likely fail to give a realistic reflection of behaviour in humans. The other species differences noted earlier, such as differences in the polymerisation state of serum IgA, tend to compound this problem. The mouse is considered especially unsuitable for testing the function of human IgA because it lacks the equivalent of human FcαRI. To circumvent this issue, mice transgenic for human CD89 have been generated and used widely as useful models for analysis of the function of human IgA [157,158]. Another notable milestone in creation of useful mouse models was the generation of a human IgA knock-in mouse [159].

## 6. Current landscape of IgA-Based Therapeutics

### 6.1. Comparisons of IgG and IgA mAbs in Cancer Therapy

Traditional cancer therapies of removal surgery or radiation for elimination of tumour cells in localised tumours and chemotherapy for metastatic tumours, while effective, are very aggressive procedures. With the development of proteomic, genomic, and bioinformatics approaches, it became possible to better characterise cancer cells and identify the proteins expressed at their surface. Thus targeting of tumour cells by antibodies directed to tumour antigens, such as glycoproteins, growth factors, cluster of differentiation (CD) antigens, is now an established treatment option [160].

Of the several therapeutic antibodies used in cancer treatment, some are used in solid tumours, targeting specific antigens such as the epidermal growth factor receptor (EGFR) found in colorectal cancer, or the human epidermal growth factor 2 (HER2) associated with breast cancer [161]. More “liquid” tumours such leukaemias and lymphomas have also been successfully treated. For example, B-cell lymphomas have been treated with anti-CD20 mAbs [162]. Indeed, Rituximab, an anti-CD20 antibody, was the first monoclonal antibody approved for cancer therapy in 1997, being followed by several others, including Cetuximab (anti-EGFR) and Trastuzumab (anti-HER2), all of the IgG isotype [163]. 

These mAbs work in different ways, with anti-CD20 mAb inducing apoptosis and sensitising tumour cells for chemotherapy, anti-HER2 inhibiting intracellular pathways involved in cancer progression, and anti-EGFR binding to growth factor receptors and blocking cancer cell proliferation [164,165,166]. However, their performance will often depend on the expression levels of the antigen on the tumour cells and can be affected by mutations in downstream pathways. Being of the IgG subclass, these mAbs are able to activate the complement pathway and interact with Fcγ receptors, eliminating tumours by cell lysis or targeting tumour cells for elimination by immune cells. There has been debate regarding which subset of immune cells is more important for mAb therapy, with natural killer (NK) cells seen for a long time as the main effectors, promoting apoptosis of tumour cells [167]. Macrophages, and to a lesser extent monocytes, were also recognised for their phagocytosis ability towards tumour cells coated with antibodies [168], while neutrophils were associated with tumour regression, even in the absence of mAbs [169]. Neutrophils, besides secreting cytotoxic agents, can lead to necrotic and autophagic tumour cell death, and can be recruited in large numbers, especially upon stimulation with granulocyte-colony stimulating factor (G-CSF) and granulocyte macrophage-colony stimulating factor (GM-CSF) [170,171]. The importance of neutrophils in tumour clearance was shown in a B-cell lymphoma mice model, where anti-CD20 mAb was less effective when neutrophils were depleted [172]. Since neutrophils do not easily recognise tumour cells, the use of mAbs is important to establish this interaction. However, the high-affinity IgG Fc receptor FcγRI is only expressed in neutrophils upon G-CSF stimulation, and besides the numerous side effects of the stimulation, this therapeutic strategy did not lead to significant clinical responses when using IgG mAbs [173,174,175,176]. 

IgA, together with its receptor FcαRI (CD89), create another possibility for new therapies focused on the activation of FcαRI-expressing cells. Both FcαRI and FcγRI associate with FcR γ chain, but FcαRI may create stronger electrostatic interactions with the FcR γ chain promoting a more stable interaction [177]. Besides, binding to FcαRI promotes release of leukotriene B4 (LTB4), which acts as a chemoattractant for neutrophils. Therefore, targeting this receptor leads to additional neutrophil migration to tumour sites [80]. Although FcαRI expression in neutrophils is lower than that of Fcγ receptors naturally expressed in these cells (FcγRIIa and FcγRIIIb), binding of IgA or IgG to neutrophils is similar, which suggests a more stable binding by IgA and a higher efficiency at triggering neutrophils than IgG [178]. For instance, the use of an IgA anti-Ep-CAM mAb was shown to kill colon carcinoma cells, unlike the IgG1 mAb counterpart [179]. Similar results were shown for the anti-EGFR mAb, with the IgA being superior at recruiting polymorphonuclear cells than the IgG subtype [180]. 

Another alternative to target FcαRI consists in the use of bispecific antibodies (BsAb). By virtue of combining two distinct antigen binding capabilities, BsAb are able to target tumours and recruit immune cells, such as neutrophils, leading to tumour cell killing by antibody-dependent cellular cytotoxicity mechanisms [181]. The use of a BsAb against both HER2 and FcαRI (namely anti-HER2 × FcαRI) efficiently eliminated breast carcinoma cells by neutrophil accumulation, unlike the equivalent FcγRI-directed BsAb (anti-HER2 × FcγRI) [182]. The same was observed for CD20 antibodies, where IgG Abs or FcγRI and FcγRIII-directed BsAbs (anti-CD20 × FcγRI or FcγRIII) showed no ability to kill malignant B cells, whereas the equivalent FcαRI BsAb promoted malignant B cell killing via neutrophil activation [183]. Another study showed that the BsAb anti-HLA II × FcαRI was effective in recruiting polymorphonuclear cells against human B cell malignancies [184].

For a long time, in vivo studies on IgA and FcαRI cancer therapies were impaired by the lack of FcαRI in mouse. However, the development of FcαRI transgenic mice has overcome that barrier [157]. Additionally, the study of mouse IgAs in interaction with FcαRI has been hampered due to the poor binding of mouse IgA to the human FcαRI, but the knock-in of human IgA into mice (Cα1 gene knock-in) has made possible the generation of antigen-specific human IgA mAbs in mice [159]. The use of these animal models showed that anti-CD20 IgA mAbs can effectively prevent B cell lymphoma development by recruiting FcαRI-expressing immune cells [67,185]. Likewise, IgA2 anti-EGFR was proved to be more efficient than Cetuximab (IgG format) against tumour cells in a FcαRI transgenic mice model [138]. In addition to the anti-tumour response of IgA1 anti-HER2 mAb, it was shown that the introduction of an albumin binding domain allows the interaction with the neonatal Fc receptor (FcRn), which is used for IgG and albumin recycling in the serum, leading to an increase of the IgA half-life without compromising its anti-tumour activity in vivo [141]. As mentioned previously, the half-life of IgA can also be extended by decreasing clearance by ASGPR in the liver, which can be achieved by sialylation of the IgA glycans [138]. A higher sialylation of the *N*-glycans in the IgA anti-HER2 did not interfere in the anti-tumour response and lead to the decrease in tumour growth in FcαRI transgenic mice, while increasing the antibody half-life [140]. In another study, the removal of two glycosylation sites and two free cysteines, together with a stabilised HC and LC linkage, created a new IgA2 anti-EGFR mAb with a longer half-life than the wild-type antibody, and higher efficacy due to Fab-mediated effects and interaction with myeloid cells expressing FcαRI [139]. 

### 6.2. IgA mAbs in Treating or Preventing Infections

Several anti-infective mAbs of the IgG isotype are approved to combat infectious diseases, namely, Palivizumab against respiratory syncytial virus, Raxibacumab and Obiltoxaximab against anthrax, and Bezlotoxumab to combat *Clostridium difficile* [186].

As the most abundant antibody at the mucosal surfaces, IgA has the important role of detecting and alerting the immune system to pathogens, whilst not responding to commensal bacteria and environmental antigens, representing an important means to combat infectious diseases. IgA antibodies were shown to be effective against tuberculosis infection in a mouse model. The passive intranasal inoculation with a mouse IgA mAb against the α-crystallin antigen of *Mycobacterium tuberculosis* led to a significant decrease in bacteria in the lungs, when either monomeric or polymeric forms of the antibody were used. Despite the transitory protective effect, probably due to the fast degradation of the administered IgA, this antibody was shown to combat early infection in the lungs, with potential use for immunoprophylaxis in immunocompromised individuals at risk of tuberculosis infection [187]. In a later study, the use of a human IgA1 against *M. tuberculosis* showed that the protective effect of the passive inoculation is dependent on the presence of FcαRI, being observed only in mice transgenic for human FcαRI [188]. These results suggest that the interaction between the human IgA1 and FcαRI on neutrophils and macrophages allows binding and elimination of *M. tuberculosis*. In the same study, in vitro infection of human whole blood or isolated monocytes by *M. tuberculosis* was reduced in the presence of specific IgA1 [188]. 

The importance of interaction with FcαRI was also shown for control of *Escherichia coli* infection, which when recognised by human serum IgA, can be efficiently phagocytised by FcαRI-expressing cells [189]. This ability of IgA to bind FcαRI and directly induce neutrophil migration was shown to be an important defense mechanism against several other bacteria, such as *Streptococcus pneumonia*, *Staphylococcus aureus*, *Porphyromonas gingivalis*, *Candida albicans*, *Bordetella Pertussis,* and *Neisseria meningitidis* [81,190,191,192,193,194].

The immune exclusion ability of IgA was also shown in the context of *Salmonella typhimurium* infection, where mice were orally challenged with the bacteria alone or the bacteria complexed with plasma-derived IgA and IgM [195]. Reduced bacteria dissemination was reported in mice exposed to the IgA/IgM immune complexes, mainly for antibodies coupled with the secretory component (SC), whilst IgG was unable to form immune complexes and consequently protect against *S. typhimurium* spread in gut immune structures [195]. Besides, oral administration of SIgA/M prior to intragastric *S. typhimurium* challenge is sufficient to protect mice from infection [196]. Despite the studies showing the potency of these IgA antibodies to prevent bacterial infections, all the existing immunoglobulin preparations used clinically for replacement therapy contain only IgG [197].

Passive immunisation with monomeric IgA can also be applied for viral infections. The use of vaccines against influenza virus showed the emergence of both IgA and IgG in nasal washes, but it was difficult to establish the importance of these antibodies individually [198,199]. Passive immunisation with IgG or pIgA by intravenous injection culminated in specific transport of these antibodies into nasal secretions [200]. However, high doses of IgG anti-influenza have to be injected in order to detect its presence in mice nasal secretions, and even higher doses are needed to decrease viral shedding [201]. On the other hand, administration of polymeric IgA at levels normally found in convalescent mice is enough to eliminate nasal viral shedding. Therefore, SIgA prevents infection of the upper respiratory tract, while serum IgG is important as a secondary response, acting at a later stage by detecting viruses that escaped IgA neutralisation and preventing lung infection [201]. A study using rotavirus showed that mice can be protected from infection when IgA mAb against the viral capsid was systemically administrated, but not when added to the intestinal lumen, showing the importance of transcytosis as a way of viral inactivation [65].

Passive immunisation was also tested on simian models of HIV infection. Intrarectal administration of IgG and dimeric IgA specific for the viral envelope showed that dimeric IgA provided the best protection in vivo upon SHIV infection in rhesus monkeys [202]. The protection conferred by dimeric IgA was suggested to be related to its ability to directly neutralise the virus and to form complexes that prevented free viruses crossing the epithelial cell layer. Based on the interaction of SIgA with mucosal microfold (M) cells, another study explored the transport of an HIV antigen for immunisation via this mechanism. SIgA bound to the HIV antigen was delivered orally and transported across the epithelial barrier to be captured by dendritic cells, starting mucosal and systemic immune responses that ultimately showed to be protective against infection by a recombinant virus expressing the HIV antigen [203]. Therefore, infection can be impaired by several IgA associated mechanisms, either by immune exclusion, intracellular inactivation, or recognition and activation of the immune system.

### 6.3. FcαRI Blocking Agents

Targeting FcαRI can be used as a strategy to combat autoimmune diseases, to inhibit IgG-induced phagocytosis or IgE-mediated allergic diseases. In autoimmune diseases, binding of IgA to FcαRI leads to enhanced activation of immune cells, and therefore, blocking this interaction can be beneficial to decrease tissue damage. The exposure of neutrophils to IgA immune complexes obtained from rheumatoid arthritis patients leads to in vitro release of neutrophil extracellular traps, which consist of web-like structures made of DNA and proteins that, despite capturing pathogens, are associated with tissue damage. However, the use of an anti-FcαRI mAb (MIP8a) was shown to successfully decrease neutrophil extracellular traps formation [204]. The same anti-FcαRI mAb was shown to prevent IgA autoantibodies inducing tissue damage in an ex vivo human skin model for linear IgA bullous disease [97]. Beyond mAbs, peptides that bind to the interaction sites of IgA and FcαRI could also inhibit IgA-induced neutrophil migration, having the advantage to be able to penetrate into the skin, which opens up the possibility of using them for skin autoimmune disease therapy [96]. 

Besides IgA, other antibodies can start immune responses that, when exacerbated, can be harmful, culminating in extensive inflammation or allergies. Binding of FcαRI by monomeric IgA is known for its anti-inflammatory nature through ITAMi signalling in effector cells [205]. Therefore, the IgA–FcαRI interaction can be explored as a tool to alleviate inflammation and further tissue damage caused by other antibodies. Using an allergy mice model, it was possible to show a decrease in airway inflammation upon crosslinking of FcεRI with IgE immune complexes in a FcαRI transgenic mice treated with the anti-FcαRI mAb A77 [206]. In another study, monomeric IgA was shown to successfully abrogate arthritis in a FcαRI transgenic mice model where IgG anti-collagen was used to cause rheumatoid arthritis [207]. Using a FcαRI transgenic mice model with glomerulonephritis and obstructive nephropathy caused by accumulation of IgG immune complexes, the Fab A77 targeting FcαRI was shown to be able to suppress inflammation [208]. It was also established that renal inflammation induced by different agents can be alleviated by the use of Fab fragments that target FcαRI (MIP8a) or monomeric IgA [209,210]. Therefore, targeting FcαRI either through IgA binding or the use of specific antibodies, can be used as a strategy to initiate anti-inflammatory responses in inflammatory diseases that involve myeloid cells.

## 7. Summary and Conclusions

The structural features of IgA impart this Ab class with unique functional capabilities, which are yet to be fully harnessed for therapeutic benefit. Increasing numbers of mAbs have been approved for clinical use in the last few years, and many more are currently undergoing clinical trial [211,212]. Recent examples tend to be humanised or fully human, but invariably of the IgG isotype. To date, no antibodies of the IgA isotype are known to be going through clinical trials. Regarding BsAbs, only a very few have been approved for use in the United States, while several await approval or are in preclinical and clinical trials [213]. In this context, FcαRI-targeting BsAbs are yet to reach this stage, indicating that further effort is required before the potential of IgA/FcαRI related therapies can be realised. As that point approaches, interest will undoubtedly turn to options for delivery to mucosal sites. Progress with topical application of nebulised Igs in the lungs of experimental animals [214,215] suggest that suitable strategies for mucosal delivery of mAbs in humans may appear, and we can anticipate that IgA-based mAbs will emerge as an important new arm of the arsenal of therapeutic mAbs.
